# Patient eligibility for amyloid-targeting immunotherapies in Alzheimer's disease

**DOI:** 10.1016/j.tjpad.2025.100102

**Published:** 2025-02-25

**Authors:** Jurij Rosen, Frank Jessen

**Affiliations:** a Department of Psychiatry, Faculty of Medicine and University Hospital Cologne, University of Cologne, Cologne, Germany; b Excellence Cluster on Cellular Stress Responses in Aging-Associated Diseases (CECAD), University of Cologne, Cologne, Germany; c German Center for Neurodegenerative Diseases (DZNE), Bonn, Germany

**Keywords:** Alzheimer's disease neuropathological changes, Aducanumab, Lecanemab, Donanemab

## Abstract

**Background:**

Amyloid beta (Aβ) targeting immunotherapies have evolved as promising treatment options for patients with early symptomatic Alzheimer's disease (AD). Understanding how eligibilty criteria impact on the number of patients potentially qualifying for treatment is of high relevance for designing diagnostic workflows in clinical practice and for estimating required ressources and costs.

**Objectives:**

We aimed at estimating the number of potentially eligible patients for treatment with the Aβ targeting antibodies aducanumab, lecanemab and donanemab in a specialized center real-world sample by the applying the phase 3 clinical trial and the appropriate use recommendations (AUR) inclusion and exclusion criteria to the data set. The post-mortem report was used for defining amyloid positivity and the presence of AD pathology in this study.

**Design:**

Retrospective, descriptive study.

**Setting:**

The multicenter National Alzheimer‘s Coordinating Center-Uniform Data Set (NACC-UDS) and Neuropathology Data Set (NACC—NP).

**Participants:**

We included all 3,343 participants of the NACC dataset with available post-mortem pathology reports.

**Measurements/Results:**

887 participants were potential candidates for anti-Aβ immunotherapy as they presented with amnestic mild cognitive impairment or mild dementia and the clinical diagnosis of AD (amnestic AD syndrome). Applying the criterion of amyloid positivity (post mortem report) and the clinical trial inclusion and exclusion criteria to this sample resulted in 83 (9 %), 275 (31 %), and 172 (19 %) participants eligible for treatment with aducanumab, lecanemab, and donanemab, respectively. Applying the criteria of the AUR resulted in 242 (27 %) and 266 (30 %) participants eligible for treatment with aducanumab or lecanemab, respectively. The eligible participant groups for each antibody showed partial, but not full overlap. Co-pathologies were common.

**Conclusions:**

The number of eligible participants varies between the different antibodies and the selected groups only partly overlap, indicating partly different groups of eligible participants for each antibody. Since not all inclusion and exclusion criteria can be extracted from the NACC-UDS dataset, the real number of eligible patients will be smaller.

## 1 Introduction

Alzheimer's disease (AD) is the most common cause of dementia and one of the main causes of morbidity and mortality in an aging population [1]. Therefore, specific care and treatment of AD is of pivotal importance for both the affected patients and society as a whole [1,2]. Immunotherapy using amyloid beta (Aβ) targeting antibodies has recently evolved as a disease-modifying therapy (DMT) in patients with early symptomatic AD [3]. Based on two phase 3 trials with inconsistent clinical results (both terminated after futility analysis) aducanumab was the first antibody to receive accelerated approval as a DMT for early AD by the United States Food and Drug Administration (FDA) in 2021. The accelerated approval was based on amyloid removal, which was interpreted as reasonable likely to confer clinical benefit [3]. In 2024, commercialization of aducanumab was terminated worldwide and studies to generate sufficient clinical data for full FDA approval were stopped. The application for marketing authorisation in the European Union (EU) was withdrawn by the company before evaluation by the European Medicines Agency (EMA). The antibodies lecanemab and donanemab both received full approval for the treatment of early AD by the FDA in 2023 and 2024 based on phase 3 clinical data showing amyloid removal and significant clinical efficacy [4,5]. Both are also licensed in China and Japan. Lecanemab is additionally approved in South Korea, Israel, and the United Arab Emirates (UAE). In the United Kingdom (UK) both are approved with the exclusion of homozygote carrieres of the 4-allele of the apolipoprotein E gene (APOE 4/4). Both are still under evaluation in the EU.

The trial populations of the phase 3 clinical studies of these antibodies were rigorously defined by clinical and neuropsychological inclusion and exclusion criteria as well as the in-vivo evidence of amyloid on positron-emission tomography (PET) or by cerebrospinal fluid (CSF) testing [[4], [5], [6]]. Given the assumption that immunotherapy is most effective in the early symptomatic stage of AD, namely mild cognitive impairment (MCI) and mild dementia, and the fact that amyloid-related imaging abnormalities (ARIA) are side-effects of concern, treatment in clinical care is recommended in patients, who largely match those included in phase 3 clinical trials.

While the prescribing information of the antibodies approve the use for a wider range of patients, United States (US) dementia experts published appropriate use recommendations (AUR) for the respective compounds in order to achieve an optimal benefit/risk ratio for patients in clinical practice [7,8]. The AUR provide extensive guidance on several aspects of clinical use of these antibodies including, but not limited to, indication, communication with patients about the treatment, application, safety monitoring and side effect management. The inclusion and exclusion criteria provided by the AUR largely adopt those from the clinical trials [7,8]. In addition, the AUR propose exclusion criteria based on the safety findings from the clinical trials [3,7,8]. Until now, AUR are available for aducanumab and lecanemab. The AUR for donanemab have not been published yet.

Given the costs of the treatment and monitoring, there is a substantial concern in many countries about the expected number of patients qualifying for treatment and the related burden on healthcare resources. So far, a few studies have been published reporting the number of eligible patients in specific settings or samples [[9], [10], [11], [12], [13], [14], [15]]. In the present study, we analyzed the The National Alzheimer‘s Coordinating Center-Uniform Data Set (NACC-UDS) and the NACC—Neuropathology (NACC—NP) Data Set, which is collected by the National Institute on Aging (NIA)-funded Alzheimer's Disease Research Centers (ADRC). It contains cross-sectional and longitudinal clinical care data of participants with cognitive impairment. The biomarker assessment in the NACC-UDS dataset is non-standardized, and the NACC steering board recommends against its the use for research purposes. The NACC—NP dataset, however, contains post-mortem data of NACC-UDS participants, which provides information on AD pathology. In this study, we applied the phase 3 clinical trial inclusion and exclusion criteria of aducanumab, lecanemab, and donanemab as well as the AUR criteria for treatment of aducanumab and lecanemab to determine how many participants of the NACC-UDS cohort would be eligible for treatment and how the participants’ groups for the different antibodies overlap. Post-mortem NACC—NP data were used to define amyloid positivity and the presence of AD pathology. In addition, we describe the co-pathologies in all groups. We chose to include aducanumab for completeness acknowledging that is not available for patient treatment at present.

## 2 Methods

### 2.1 Dataset

We obtained the publicly available NACC-UDS and NACC—NP Data Set upon our request. The procedures of the NACC-UDS and the NACC—NP have been described in detail before [[16], [17], [18]]. In brief, participants are referred to an ADRC by clinicians, by themselves, by their family members, or have reached the site through active community recruitment according to the procedures of each individual ADRC. At each ADRC, the participants’ data are collected with a standard protocol [16,17,19] by clinicians and trained interviewers. Post-mortem neuropathological evaluations are conducted at each ADRC. Written informed consents are obtained from participants at each ADRC and approved by the ADRC's Institutional Review Board. Research using the NACC database is approved by the University of Washington Institutional Review Board. This analysis used data from 41 ADRCs.

### 2.2 Data selection

Participants enrolled in the NACC-UDS between June 2005 and August 2021 were the basis for the present analyses. Demographic data, the Mini-Mental-State-Examination (MMSE) score, the Clinical Dementia Rating (CDR) global score according to the CDR® Dementia Staging Instrument plus NACC FTLD Behavior & Language Domains, and information on comorbidities and medication at the baseline visit were used as participants’ characteristics. The post-mortem reports, which were used to determine the presence or absence of AD pathology, include the Thal phases of Aβ/amyloid plaques, the Braak neurofibrillary tangles (NFT) staging, and the Consortium to Establish a Registry of AD (CERAD) neuritic plaque score. In the NACC—NP, AD pathology in total is defined according to the ABC score by summarizing the Thal phase (A), the Braak stage (B), and the CERAD neuritic plaque score (C) to a summary score. The score can be classified as “high”, “intermediate”, “low”, or “not” as described in more detail previously [20]. The ABC scores “high” and “intermediate” are interpreted as the presence of AD pathology, while the scores “low” and “not” are interpreted as the absence of AD pathology. According to the NACC—NP reports on co-pathologies, information on Lewy body pathology, hippocampal sclerosis, infarctes and lacunes, single and multiple old hemorrhages, fronto-temporal lobe degereration (FTLD) with tau pathology, FTLD-tau Pick's disease subtype, and other tau-pathologies was used.

### 2.3 Application of treatment eligibility criteria

Of all participants with an available post-mortem pathology report, those with a documented “amnestic AD syndrome” at the baseline visit were selected. The amnestic AD syndrome was defined by (i) cognitive impairment corresponding to mild cognitive impairment (MCI) or mild dementia operationalized by a global CDR score [21,22] of 0.5 or 1, respectively, (ii) at least mild memory impairment according to the CDR® plus NACC FTLD, and (iii) the physician's rating that the symptoms indicate AD as the most likely etiological diagnosis according to the National Institute of Neurological and Communicative Diseases and Stroke / Alzheimer's Disease and Related Disorders Association or NIA-Alzheimer's Association criteria for AD dementia [23,24].

Inclusion criteria of the clinical phase 3 trials of aducanumab (ENGAGE/EMERGE), lecanemab (CLARITY-AD), and donanemab (TRAILBLAZER-ALZ 2) were applied to the participants’ datasets [[4], [5], [6],^8^]. Supplement Tables 1, 3 and 5 show how the application was operationalized. The same approach was taken for the AUR of aducanumab and lecanemab (operationalization see supplement Tables 2 and 4) [7,8]. Given the information available in the NACC-UDS, not all criteria of the trials and the AUR could be mapped.

To operationalize the trial criterion of amyloid positivity on PET, we referred to studies that reported post-mortem Thal phase of 3–5 to correspond to a positive amyloid-PET in the living human [[25], [26], [27]]. Consequently, we consider participants of the NACC cohort as amyloid positive, who showed a post-mortem Thal phase of 3–5. In addition, we determined the number of all post-mortem amyloid-positive cases, as defined by a Thal phase >0.

The Tau-PET criterion of the donanemab trial TRAILBLAZER-ALZ 2 was operationalized by a post-mortem Braak stage of 3 or 4 indicating intermediate tau spread.

In the last step, exclusion criteria of the trials and of the AUR, respectively, were applied (see supplement Tables 1–5 for operationalization) [[4], [5], [6], [7], [8]].

### 2.4 Data analysis

For each trial and AUR, the number of participants fulfilling the clinical inclusion and exclusion criteria were determined. We also counted the number of cases with an CDR global score of either 0.5 or 1 and the number of participants with full AD pathology in each group. For each group, the co-pathologies were additionally counted. Euler diagrams were created to visualize the sample sizes and the overlap between the respective groups. For data analysis and creation of figures, the statistical computing language and environment R was used [[28], [29], [30]].

## 3 Results

### 3.1 Selection of participants based on clinical trial criteria

Of 44,359 participants included in the NACC-UDS, 3,343 participants had available post-mortem pathology reports (mean interval between baseline visit and death: 6.8 years, standard deviation (SD): 3.4 years). Of these, 887 participants showed an amnestic AD syndrome at baseline. Of these, 153 (17 %) fulfilled the clinical inclusion criteria for the aducanumab trials (EMERGE/ENGAGE) of which 136 (89 %, 15 % of the full sample) were amyloid positive. 407 (46 %) fulfilled the clinical inclusion criteria for the lecanemab trial (CLARITY-AD) of which 363 (89 %, 41 % of the full sample) were amyloid positive. 425 (48 %) fulfilled the clinical inclusion criteria for the donanemab trial (TRAILBLAZER-ALZ 2) of which 387 (91 %, 44 % of the full sample) were amyloid positive. After applying the exclusion criteria of the clinical trials, 83 participants (9 % of the full sample) remained eligible for aducanumab, 275 (31 % of the full sample) for lecanemab, and 172 (19 % of the full sample) for donanemab. After the application of the tau spread criterion of TRAILBLAZER-ALZ 2 (Braak stage 3–4), only 8 participants (1 % of the full sample) remained eligible for donanemab, while 94 % were in Braak stage 5–6. Given the suspected spread of tau over time between the baseline visit and autopsy, we did not consider this criterion further.

Of the selected participants, 81 (98 %) of the aducanumab group, 269 (98 %) of the lecanemab group, and 170 (99 %) of the donanemab group showed full AD pathology according to the ABC criteria. When including all amyloid-positive participants defined by a Thal phase >0, the number of eligible participants for aducanumab, lecanemab, and donanemab increased by 8 (10 %), 16 (6 %), and 8 (5 %) respectively.

**Fig. 1:**
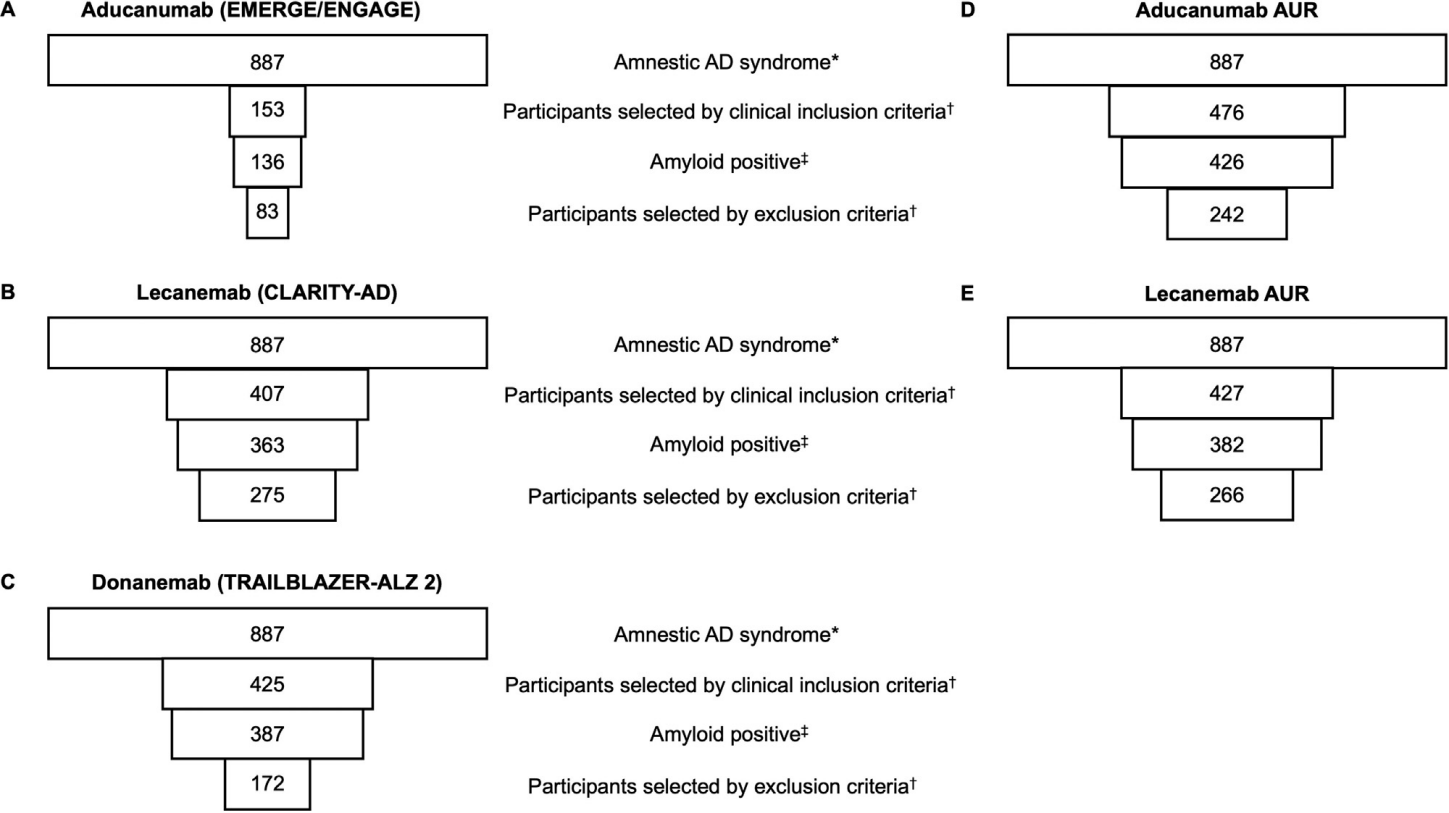
Number of participants in The National Alzheimer‘s Coordinating Center-Uniform and Neuropathology Data Set after each selection step. The width of the rectangles represents the relation of number of participants at each step. * Mild Cognitive Impairment or mild dementia (according to a global Clinical Dementia Rating score of 0.5 or 1, respectively) with at least a mild memory impairment and AD as the most likely etiological diagnosis. ^†^ Inclusion and exclusion criteria according to the respective phase 3 clinical trial criteria / AUR. ^‡^ Thal phase 3–5 at autopsy. Abbreviations: AD = Alzheimer's disease, AUR = Appropriate Use Recommendations.

**Fig. 2:**
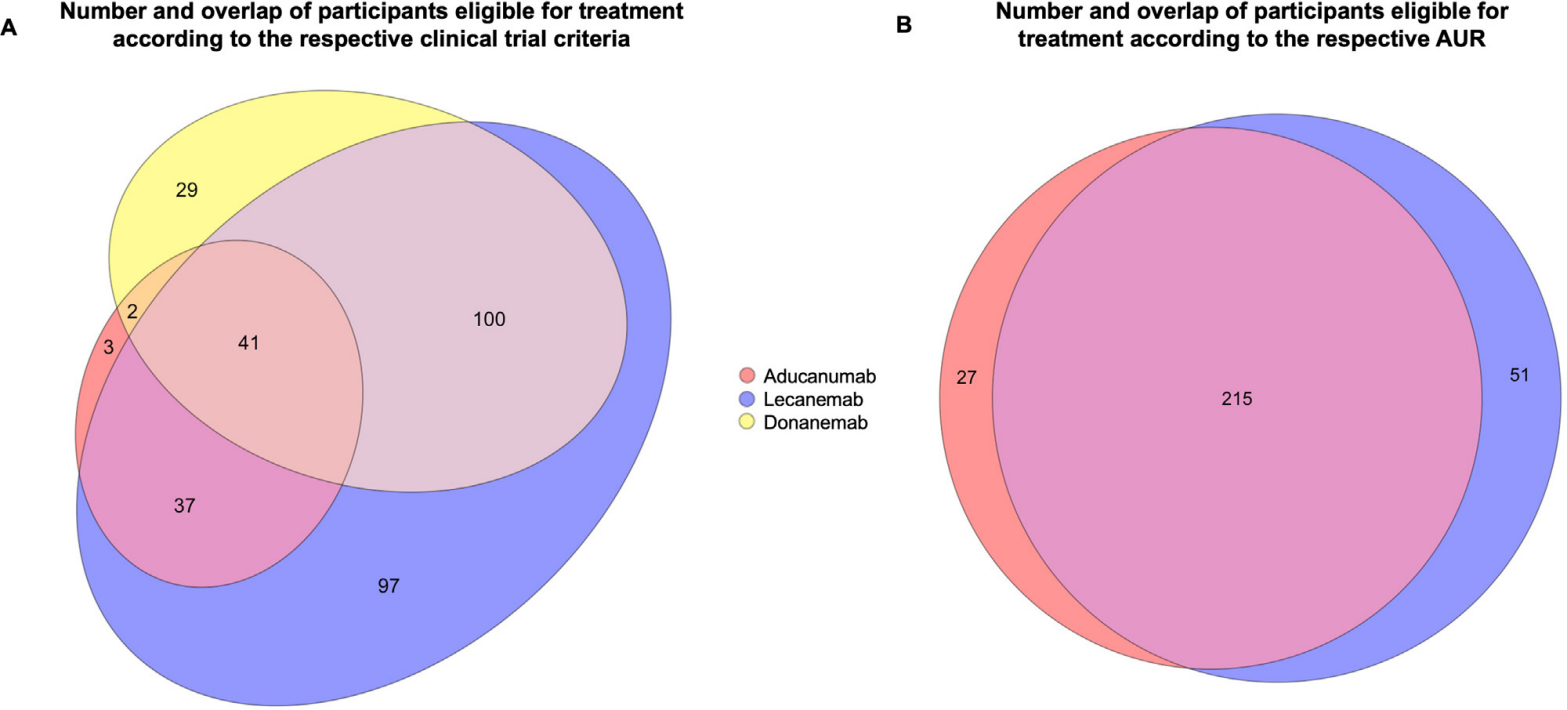
Number and overlap of eligible participants after mapping the phase 3 clinical trial criteria (A) and the AUR (B) of the indicated antibodies (colored) onto The National Alzheimer‘s Coordinating Center-Uniform Data Set and Neuropathology Data Set. The size of the ellipses and circles reflects the respective sample sizes with overlap. AUR = Appropriate Use Recommendations.

The proportion of participants with a CDR global score of 0.5 differed between the samples (100 % for aducanumab, 50 % for lecanemab and 47 % for donanemab). Co-pathologies of the selected participants are listed in Table 1. Fig. 1 shows the selection process and the resulting samples. The overlap of the samples is depicted in Fig. 2.

**Table 1:** Co-pathologies in participants fulfilling inclusion and exclusion criteria of the clinical trials or the appropriate use recommendations (AUR).

	Clinical trials			AUR	
Co-pathology (%)	Aducanumab (*n* = 83)	Lecanemab (*n* = 275)	Donanemab (*n* = 172)	Aducanumab (*n* = 242)	Lecanemab (*n* = 266)
Lewy body pathology	52	49	53	52	52
Hippocampal sclerosis	16	19	20	17	19
Infarcts and lacunes	11	10	9	11	11
Single/multiple old hemorrhages	2	1	2	1	2
FTLD with tau pathology or other taupathy	4	5	3	7	6
FTLD-tau subtype – Pick's disease	0	0	1	0	0

Results are indicated as percent (%). The proportion of participants in whom the co-pathologies was not reported was low (≤ 2 %). Clinical trials: EMERGE/ENGAGE (aducanumab), CLAIRITY-AD (lecanemab), TRAILBLAZER-ALZ 2 (donanemab).

### 3.2 Selection of participants based on the appropriate use recommendations (AUR)

Of 887 participants with an amnestic AD syndrome at baseline, 476 (54 %) fulfilled the clinical inclusion criteria of the aducanumab AUR of which 426 (89 %, 48 % of the full sample) were amyloid positive, and 427 (48 %) fulfilled the clinical inclusion criteria for lecanemab of which 382 (89 %, 43 % of the full sample) were amyloid positive. After applying the exclusion criteria, 242 (27 % of the full sample) were eligible for aducanumab, and 266 (30 % of the full sample) for lecanemab. Of the selected participants, 239 (99 %) of the aducanumab group, and 261 (98 %) of the lecanemab group showed full AD pathology according to the ABC criteria. When including all amyloid-positive participants defined by a Thal phase >0, the number of participants eligible for aducanumab and lecanemab increased by 17 (7 %), and 16 (6 %) respectively.

**Fig. 3:**
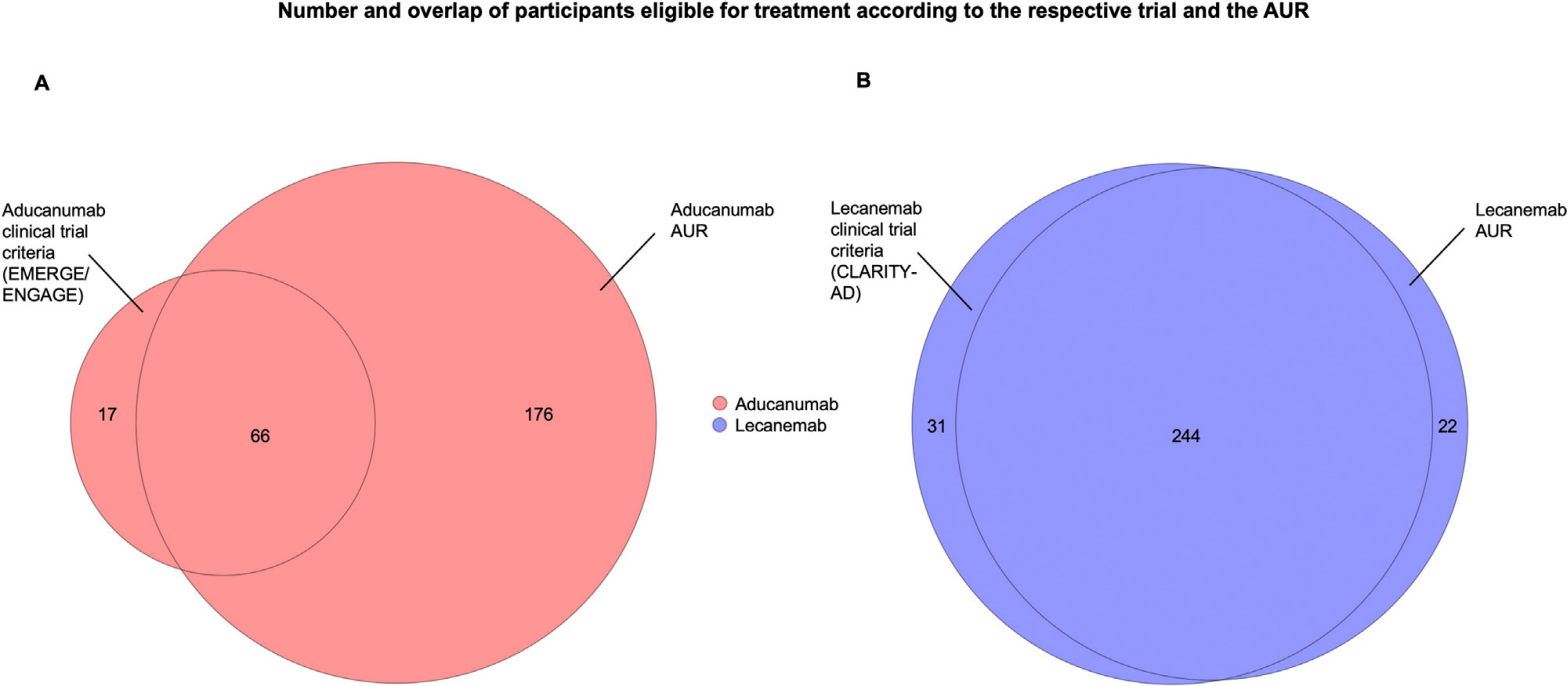
Number and overlap of eligible participants after mapping the phase 3 clinical trial criteria and the AUR of the indicated antibody (colored) onto The National Alzheimer‘s Coordinating Center-Uniform Data Set and Neuropathology Data Set. The size of the circles reflects the respective sample sizes. AUR = Appropriate Use Recommendations.

In both groups, the proportion of participants with a CDR global score of 0.5 was similar (50 % and 51 % for the aducanumab and lecanemab samples, respectively). Co-pathologies of the selected participants are listed in Table 1. Fig. 1 shows the selection process and the resulting samples. Fig. 2 shows the overlap of the selected groups. Fig. 3 shows the overlap of participants who are eligible for treatment with aducanumab or lecanemab according to the clinical trial criteria and the AUR.

## 4 Discussion

Applying the selection criteria of the clinical trials or the AUR of current amyloid-targeting antibodies to participants with an amnestic syndrome at the stage of MCI or mild dementia (CDR global score of 0.5 or 1) in our study, results in a maximum of 30 % who would eventually be eligible for treatment. Our findings are consistent with the results of previous studies, which have indicated that the majority of patients in the early stages of symptomatic AD do not qualify for immunotherapy. In more detail, the study by Pittock et al. examined the number of eligible participants according to the clinical trial selection criteria of lecanemab and aducanumab in participants of the Mayo Clinic Study of Aging, a population of 5,255 community-dwelling research volunteers [9]. This study found that among the subset of 237 participants with MCI or mild dementia and increased brain amyloid burden, only 8 % were eligible for lecanemab and 5.1 % for aducanumab. Rosenberg et al. determined the eligibility for treatment with aducanumab according to the AUR in the single center memory clinic population of Karolinska University Hospital [10]. The analysis comprised 410 participants with cognitive complaints, including subjective cognitive impairment, MCI and dementia. The authors found that only approximately 10 % of participants met the eligibility criteria for treatment, with some variation depending on different biomarker cutoffs. Likewise, other studies have examined eligibility for immunotherapy by applying the selection criteria of the clinical trials or the AUR of either antibody to patient data from memory clinics and found comparable results [[11], [12], [13], [14]]. Moreover, based on National Health Service data, Laurell et al. estimated that 30,200 patients (3.1 % of all dementia cases) would qualify for antibody treatment in the United Kingdom [15].

While the cited studies all used biomarkers (CSF or PET) for determining amyloid, we used post-mortem data. The advantage of our approach is that the post-mortem examination can be considered the gold standard for detection of pathology. It also provides quantitative and spatial information of amyloid below the detection threshold of biomarkers. In our analysis, we first applied a Thal phase of 3 or higher as the criterion for amyloid positivity, which corresponds to a positive amyloid PET [26,27]. In a second step, we defined all cases with a Thal phase of >0 as amyloid positive, thereby including participants who would not be amyloid positive on PET. Importantly, we found that the increase by including cases with a Thal phase 1–2 was only 10 % or less in all analyses, suggesting that the proportion of minimally amyloid positive cases that are missed by biomarker criterion in the living human is small. Also, we found that 98–100 % of all participants fulfilling the clinical trial or the AUR criteria also fulfilled the ABC criteria for full AD pathology including the required tau pathology, reassuring that the amyloid criterion reflects full AD in just about all cases. The disadvantage of our approach is the interval between the baseline assessment, at which the clinical inclusion and exclusion criteria were obtained, and the post-mortem analyses, which was on average 6.8 years later. While amyloid is considered to be at a plateau before symptom onset and throughout the disease course, tau pathology is spreading during the symptomatic phase of the disease. This explains why only 1 % of all cases selected based on clinical criteria at baseline showed a Braak stage of 3–4 at later autopsy (corresponding to the tau-PET inclusion criteria in the TRAILBLAZER-ALZ 2 trial) and 94 % were at Braak stage 5–6. As such, our approach cannot be used to determine the number of participants, who fulfill the TRAILBLAZER-ALZ 2 tau criteria.

Our data show that among participants eligible for treatment, co-pathologies are common. This particularly applies to Lewy body pathology which was present in about 50 % of participants, but also for hippocampal sclerosis (16–20 %) potentially due to transactive response DNA binding protein (TDP)−43 pathology, and vascular lesions (10 %). These results are largely in line with earlier studies that investigated the prevalence of co-pathologies in AD patients at autopsy [31,32]. The presence of co-pathologies could influence cognitive trajectories [[32], [33], [34]], and it is not yet known to what extend co-pathologies impact on the efficacy of amyloid-targeting immunotherapies [34]. As a limitation, we cannot define the proportion of co-pathology, which developed during the time interval between the clinical assessment at the baseline visit, on which we mapped the inclusion and exclusion criteria, and the post-mortem evaluation.

Applying the different selection criteria to the NACC dataset resulted in samples of participants that did not fully overlap and varied in size. Consequently, the number of patients eligible for immunotherapy may vary depending on the antibody used. Moreover, given that these samples only partially overlap, the respective antibody therapies will be provided to slightly different patient groups, if treatment initiation adheres to the respective criteria. This may partially limit the comparability of the effects and side effects of these antibodies in clinical practice.

Our study demonstrates the significant difference of applying the clinical trial criteria versus the AUR in the case of aducanumab, which are primarily driven by the fact that the EMERGE and ENGAGE trials only included patients with a global CDR score of 0.5, while the AUR do not require this particular CDR score. In contrast, the number of participants selected by trial criteria and the AUR for lecanemab did not differ considerably.

Our results are limited by the fact that, while the NACC dataset represents a real-world sample of participants referred to an ADRC, its characteristics may not fully align with those of other specialized institutions. Also, neuropathological information, notably on amyloid pathology, is unavailable in clinical practice, where it is estimated by biomarkers. The available biomarkers in the NACC dataset are in turn not recommended for scientific purposes by the NACC steering board. Due to these differences, our estimates might be slightly different from those derived from a biomarker-based clinical setting. In addition, not all selection criteria from the clinical trials or the AUR could be applied to the dataset, as certain criteria were either not precisely detailed or only partially itemized (refer to supplemental Tables 1–5). This limitation was especially evident for MRI data, as information on brain pathologies observed on MRI was mostly unavailable in the NACC dataset. Consequently, the percentage of eligible patients for treatment in clinical settings might be even smaller than the present data suggest.

In conclusion, the application of different selection criteria used in phase 3 clinical trials or the AUR leads to a variable number of patients eligible for each of the different immunotherapies. Furthermore, depending on the applied selection criteria of the clinical trial, and to a lesser extent on the respective AUR, the selected patient groups only partially overlap, which needs to be considered when comparing real world treatment data.

## Funding

None.

## Declaration of generative AI and AI-assisted technologies in the writing process

During the preparation of this work the authors used OpenAI's ChatGPT in order to refine readability of the manuscript. No content generation was performed by the tool. After using this tool, the authors reviewed and edited the content as needed and take full responsibility for the content of the publication.

## CRediT authorship contribution statement

**Jurij Rosen:** Writing – review & editing, Writing – original draft, Visualization, Methodology, Investigation, Formal analysis, Data curation. **Frank Jessen:** Writing – review & editing, Validation, Supervision, Project administration, Methodology, Investigation, Formal analysis, Conceptualization.

## Declaration of competing interest

The authors declare the following financial interests/personal relationships which may be considered as potential competing interests:

Frank Jessen reports a relationship with Abbvie, AC immune, Eisai, Eli Lilly, GE Healthcare, Grifols, Janssen Cilag, Roche that includes: consulting or advisory, funding grants, and speaking and lecture fees. Jurij Rosen has received a travel grant from Eisai. If there are other authors, they declare that they have no known competing financial interests or personal relationships that could have appeared to influence the work reported in this paper.

## Appendix Supplementary materials

Image, application 1

## References

[1] (2024). 2024 Alzheimer's disease facts and figures. Alzheimers Dement.

[2] Wong W. (2020). Economic burden of Alzheimer disease and managed care considerations. Am J Manag Care.

[3] Heneka M.T., Morgan D., Jessen F (2024). Passive anti-amyloid beta immunotherapy in Alzheimer's disease-opportunities and challenges. Lancet.

[4] van Dyck C.H., Swanson C.J., Aisen P., Bateman R.J., Chen C., Gee M. (2023). Lecanemab in early Alzheimer's Disease. N Engl J Med.

[5] Sims J.R., Zimmer J.A., Evans C.D., Lu M., Ardayfio P., Sparks J. (2023). Donanemab in early symptomatic Alzheimer disease: the TRAILBLAZER-ALZ 2 randomized clinical trial. JAMA.

[6] Budd Haeberlein S., Aisen P.S., Barkhof F., Chalkias S., Chen T., Cohen S. (2022). Two randomized phase 3 studies of Aducanumab in early Alzheimer's disease. J Prev Alzheimers Dis.

[7] Cummings J., Rabinovici G.D., Atri A., Aisen P., Apostolova L.G., Hendrix S. (2022). Aducanumab: appropriate use recommendations update. J Prev Alzheimers Dis.

[8] Cummings J., Apostolova L., Rabinovici G.D., Atri A., Aisen P., Greenberg S. (2023). Lecanemab: appropriate use recommendations. J Prev Alzheimers Dis.

[9] Pittock R.R., Aakre J.A., Castillo A.M., Ramanan V.K., Kremers W.K., Jack C.R. (2023). Eligibility for anti-amyloid treatment in a population-based study of cognitive aging. Neurology.

[10] Rosenberg A., Ohlund-Wistbacka U., Hall A., Bonnard A., Hagman G., Ryden M. (2022). beta-amyloid, tau, neurodegeneration classification and eligibility for anti-amyloid treatment in a memory Clinic population. Neurology.

[11] Canevelli M., Rossi P.D., Astrone P., Consorti E., Vanacore N., Cesari M. (2021). Real world" eligibility for aducanumab. J Am Geriatr Soc.

[12] Canu E., Rugarli G., Coraglia F., Basaia S., Cecchetti G., Calloni S.F. (2024). Real-word application of the AT(N) classification and disease-modifying treatment eligibility in a hospital-based cohort. J Neurol.

[13] Padovani A., Caratozzolo S., Rozzini L., Pilotto A., Benussi A., Tedeschi G. (2022). "Real-world" eligibility for aducanumab depends on clinical setting and patients' journey. J Am Geriatr Soc.

[14] Togher Z., Dolphin H., Russell C., Ryan M., Kennelly S.P., O'Dowd S (2022). Potential eligibility for Aducanumab therapy in an Irish specialist cognitive service-utilising cerebrospinal fluid biomarkers and appropriate use criteria. Int J Geriatr Psychiatry.

[15] Laurell A.A.S., Venkataraman A.V., Schmidt T., Montagnese M., Mueller C., Stewart R. (2024). Estimating demand for potential disease-modifying therapies for Alzheimer's disease in the UK. Br J Psychiatry.

[16] Beekly D.L., Ramos E.M., Lee W.W., Deitrich W.D., Jacka M.E., Wu J. (2007). The National Alzheimer's Coordinating Center (NACC) database: the Uniform Data Set. Alzheimer Dis Assoc Disord.

[17] Morris J.C., Weintraub S., Chui H.C., Cummings J., Decarli C., Ferris S. (2006). The Uniform Data Set (UDS): clinical and cognitive variables and descriptive data from Alzheimer Disease Centers. Alzheimer Dis Assoc Disord.

[18] Weintraub S., Salmon D., Mercaldo N., Ferris S., Graff-Radford N.R., Chui H. (2009). The Alzheimer's Disease Centers' Uniform Data Set (UDS): the neuropsychologic test battery. Alzheimer Dis Assoc Disord.

[19] Besser L., Kukull W., Knopman D.S., Chui H., Galasko D., Weintraub S. (2018). Version 3 of the National Alzheimer's Coordinating Center's Uniform Data Set. Alzheimer Dis Assoc Disord.

[20] Montine T.J., Phelps C.H., Beach T.G., Bigio E.H., Cairns N.J., Dickson D.W. (2012). National Institute on Aging-Alzheimer's Association guidelines for the neuropathologic assessment of Alzheimer's disease: a practical approach. Acta Neuropathol.

[21] Hughes C.P., Berg L., Danziger W.L., Coben L.A., Martin R.L. (1982). A new clinical scale for the staging of dementia. Br J Psychiatry.

[22] Morris J.C. (1993). The Clinical Dementia Rating (CDR): current version and scoring rules. Neurology.

[23] Jack C.R., Albert M.S., Knopman D.S., McKhann G.M., Sperling R.A., Carrillo M.C. (2011). Introduction to the recommendations from the National Institute on Aging-Alzheimer's Association workgroups on diagnostic guidelines for Alzheimer's disease. Alzheimers Dement.

[24] Jack C.R., Bennett D.A., Blennow K., Carrillo M.C., Dunn B., Haeberlein S.B. (2018). NIA-AA Research Framework: toward a biological definition of Alzheimer's disease. Alzheimers Dement.

[25] La Joie R., Ayakta N., Seeley W.W., Borys E., Boxer A.L., DeCarli C. (2019). Multisite study of the relationships between antemortem [(11)C]PIB-PET centiloid values and postmortem measures of Alzheimer's disease neuropathology. Alzheimers Dement.

[26] Thal D.R., Beach T.G., Zanette M., Lilja J., Heurling K., Chakrabarty A. (2018). Estimation of amyloid distribution by [(18)F]flutemetamol PET predicts the neuropathological phase of amyloid beta-protein deposition. Acta Neuropathol.

[27] Salloway S., Gamez J.E., Singh U., Sadowsky C.H., Villena T., Sabbagh M.N. (2017). Performance of [(18)F]flutemetamol amyloid imaging against the neuritic plaque component of CERAD and the current (2012) NIA-AA recommendations for the neuropathologic diagnosis of Alzheimer's disease. Alzheimers Dement (Amst).

[28] R Core Team (2024). https://www.R-project.org/.

[29] Wickham H., Averick M., Bryan J., Chang W., McGowan L., François R. (2019). Welcome to the tidyverse. J Open Source Software.

[30] eulerr: area-Proportional Euler and Venn Diagrams with Ellipses. R package version 7.0.0 [Internet]. 2022. https://CRAN.R-project.org/package=eulerr.

[31] Hamilton R.L. (2000). Lewy bodies in Alzheimer's disease: a neuropathological review of 145 cases using alpha-synuclein immunohistochemistry. Brain Pathol.

[32] Serrano-Pozo A., Qian J., Monsell S.E., Frosch M.P., Betensky R.A., Hyman B.T. (2013). Examination of the clinicopathologic continuum of Alzheimer disease in the autopsy cohort of the National Alzheimer Coordinating Center. J Neuropathol Exp Neurol.

[33] Kraybill M.L., Larson E.B., Tsuang D.W., Teri L., McCormick W.C., Bowen J.D. (2005). Cognitive differences in dementia patients with autopsy-verified AD, lewy body pathology, or both. Neurology.

[34] Savica R., Beach T.G., Hentz J.G., Sabbagh M.N., Serrano G.E., Sue L.I. (2019). Lewy body pathology in Alzheimer's disease: a clinicopathological prospective study. Acta Neurol Scand.

