# Supplementary material for: Patient eligibility for amyloid-targeting immunotherapies in Alzheimer's disease
Source: J Prev Alzheimers Dis. 2025 Feb 25;12(4):100102. doi: 10.1016/j.tjpad.2025.100102 (PMC12184045; doi:10.1016/j.tjpad.2025.100102)
Supplement: Supplementary file 1 [file mmc1.docx]

**SUPPLEMENT TABLES**

**Table 1:** Operationalization of the mapping of selection criteria of the aducanumab trials on the NACC dataset

| Trial criteria | Operationalization the NACC database | No. of participants not fulfilling criterion |
| --- | --- | --- |
| **Inclusion criteria** |  |  |
| Ability to understand the purpose and risks of the study and provide signed informed consent | NA | - |
| Aged 50 to 85 years old, inclusive, at the time of informed consent | Age at initial visit 50-85 years (inclusive) | 80 |
| All women of childbearing potential and all men must practice highly effective contraception | NA | - |
| Must have at least 6 years of education or work experience | At least 6 years of education | 4 |
| Must have a positive amyloid PET scan | NA | - |
| CDR global score of 0.5 | Global CDR = 0.5 | 543 |
| MMSE score between 24 and 30 | Total MMSE score 24-30 (inclusive) | 104 |
| Apart from a clinical diagnosis of early AD, the subject must be in good health | Precise operationalization not possible, relevant diagnoses were excluded (see below) | - |
| Must consent to APOE genotyping | NA | - |
| Has one informant/care partner who has frequent and sufficient contact with the subject | Co-participant available | 3 |
| **Exclusion criteria** |  |  |
| Any uncontrolled medical or neurological/neurodegenerative condition (other than AD) that might be a contributing cause of the subject’s cognitive impairment (e.g., substance abuse, vitamin B12 deficiency, abnormal thyroid function, stroke or other cerebrovascular condition, Lewy body dementia, frontotemporal dementia, head trauma); history of or known seropositivity for human immunodeficiency virus; recent clinically significant systemic illness or serious infection (e.g., pneumonia, septicemia); history/ evidence of an autoimmune disorder or requiring chronic use of systemic corticosteroids or other immunosuppressants; history/ evidence of hepatitis B/C infection; other medical conditions (e.g., renal disease) that are not stable or controlled; any medications that may contribute to cognitive impairment, put the subject at higher risk for AEs, or impair the subject’s ability to perform cognitive testing or complete study procedures | Medical/neurological/ neurodegenerative condition primary or contributing cause of cognitive impairment (ie, alcohol or other substance abuse; systemic disease/medical illness; cerebrovascular disease; vascular brain injury; stroke; probable vascular dementia (NINDS/AIREN criteria); Lewy body disease; multiple system atrophy; primary supranuclear palsy; corticobasal degeneration; frontotemporal lobar degeneration (with motor neuron disease, behavioral variant, or not otherwise specified); primary progressive aphasia; essential tremor; Down syndrome; Huntington’s disease; Prion disease (Creutzfeldt-Jakob disease, other); traumatic brain injury; normal-pressure hydrocephalus; epilepsy; central nervous system neoplasm; human immunodeficiency virus; other neurological, genetic, or infectious condition; delirium; systemic disease/medical illness; medications) | 12 |
| Recent, clinically significant unstable psychiatric illness (e.g., uncontrolled major depression, uncontrolled schizophrenia, uncontrolled bipolar affective disorder) | Clinically significant psychiatric illness (severe depression/dysphoria; recent/active schizophrenia; recent/active bipolar disorder; moderate/severe hallucinations; moderate/severe delusions) | 7 |
| Recent transient ischemic attack or stroke or any unexplained loss of consciousness | Recent/active transient ischemic attack or stroke; traumatic brain injury with brief loss of consciousness | 2 |
| Brain MRI performed at Screening that shows evidence of any of the following: Acute or sub-acute hemorrhage; prior macrohemorrhage (defined as >1 cm in diameter on T2* sequence) or prior subarachnoid hemorrhage; greater than 4 microhemorrhages (defined as ≤1 cm in diameter on T2* sequence); cortical infarct (defined as >1.5 cm in diameter); >1 lacunar infarct (defined as ≤1.5 cm in diameter); superficial siderosis; history of diffuse white matter disease as defined by a score of 3 on the age-related white matter changes scale; any finding that might be a contributing cause of subject’s dementia, might pose a risk to the subject | Evidence of macrohemorrhage(s), microhemorrhage(s), large vessel infarct(s), lacunar infarct(s), extensive white-matter hyperintensity (Cardiovascular Health Study score 7-8) | 0 |
| History of bleeding disorder or predisposing conditions, blood clotting or clinically significant abnormal results on coagulation profile at Screening | NA | - |
| Presence of uncontrolled diabetes mellitus; history of unstable angina, myocardial infarction, chronic heart failure, or clinically significant conduction abnormalities (e.g., unstable atrial fibrillation); clinically significant 12-lead ECG abnormalities; uncontrolled hypertension defined as: average of 3 systolic blood pressure [SBP]/diastolic blood pressure [DBP] readings >165 mmHg and/or >100 mmHg, or persistent SBP/DBP readings >180 mmHg and/or >100 mmHg 3 months prior to randomization; history of malignancy or carcinoma; history of seizures | Diabetes (recent/active); angina (present); myocardial infarct (present within the past 12 months); congestive heart failure (recent/active); atrial fibrillation (recent/active); pacemaker (recent/active); blood pressure readings >165 mmHg (systolic) and/or >100 mmHg (diastolic) at the initial visit; cancer (primary/non-metastatic or metastatic); seizures (recent/active, remote/inactive) | 22 |
| Indication of impaired liver function as shown by an abnormal liver function profile | NA | - |
| Recent history of alcohol or substance abuse; use of illicit narcotic medication | Alcohol abuse – clinically significant impairment occurring over a 12-month period manifested in one of the following areas: work, driving, legal, or social (recent/active); other abused substances – clinically significant impairment occurring over a 12-month period manifested in one of the following areas: work, driving, legal, or social (recent/active) | 0 |
| History of severe allergic or anaphylactic reactions, or history of hypersensitivity to any of the inactive ingredients in the drug product | NA | - |
| Use of allowed chronic (AD-) medications at doses that have not been stable for at least 4 (8) weeks prior to Screening Visit | Data on recent change of medication is limited | - |
| Recent vaccinations | NA | - |
| Participation in any active/ passive immunotherapy study targeting Ab study with purported disease-modifying effect in AD; previous study with aducanumab | NA | - |
| Contraindications to having a brain MRI or PET scan | Participants with pacemaker excluded (see above) | - |
| A recent negative PET scan result with any amyloid-targeting ligand | NA | - |
| Have had or plan exposure to experimental radiation | NA | - |
| Contraindications to having a LP | NA | - |
| Female subjects who are pregnant or currently breastfeeding | NA | - |
| Subject currently living in an organized care facility | Participant living in nursing home | 3 |
| Use of medications with platelet anti-aggregate or anti-coagulant properties (the use of aspirin at a prophylactic dose is allowed) | Use of one of the following medications with platelet anti-aggregate or anti-coagulant properties (ie, heparin; enoxaparin; dalteparin; danaparoid; ardeparin; tinzaparin; heparin flush; warfarin; anisindione; dicumarol; lepirudin; argatroban; bivalirudin; dabigatran; fondaparinux; rivaroxaban; apixaban; dipyridamole; ticlopidine; clopidogrel; cilostazol; aspirin-dipyridamole; prasugrel; ticagrelor; abciximab; tirofiban; eptifibatide) | 7 |
| Recent blood donation | NA | - |
| Inability to comply with study requirements or other unspecified reasons that make the subject unsuitable for enrollment | NA | - |

Operationalization of inclusion and exclusion criteria of the trials (left column) onto the NACC dataset by the indicated variable (middle column). NA indicates the absence of the respective information in the dataset. For all criteria that could be mapped onto the NACC dataset, the number of participants not fulfilling the respective criteria is indicated (rightmost column). Abbreviations: NINDS/AIREN = National Institute of Neurological Disorders and Stroke / Association Internationale pour la Recherche et l'Enseignement en Neurosciences. Note that some criteria of the trial were shortened / summarized to improve readability.

**Table 2:** Operationalization of the mapping of selection criteria for treatment with aducanumab according to the AUR

| Appropriate Use Recommendations criteria | Operationalization the NACC database | No. of participants not fulfilling criterion |
| --- | --- | --- |
| **Inclusion criteria** |  |  |
| Age 50-85; younger or older patients meeting all other criteria for treatment may be considered candidates for aducanumab | Age at initial visit ≤95 years | 5 |
| Mild decline of cognition with no or limited impairment of activities of daily living established by objective cognitive testing | Total MMSE score 22-30 (inclusive) | 406 |
| Amyloid positive PET or CSF findings consistent with AD | NA | - |
| APOE genotype determined | NA | - |
| **Exclusion criteria** |  |  |
| Non-AD neurological disorders excluded | Neurological condition primary or contributing cause of cognitive impairment (cerebrovascular disease; vascular brain injury; stroke; probable vascular dementia (NINDS/AIREN criteria); Lewy body disease; multiple system atrophy; primary supranuclear palsy; corticobasal degeneration; frontotemporal lobar degeneration (with motor neuron disease, behavioral variant, or not otherwise specified); primary progressive aphasia; essential tremor; Down syndrome; Huntington’s disease; Prion disease (Creutzfeldt-Jakob disease, other); traumatic brain injury; normal-pressure hydrocephalus; epilepsy; central nervous system neoplasm; human immunodeficiency virus; other neurological, genetic, or infectious condition; delirium); recent or active transient ischemic attack/stroke/seizures/traumatic brain injury with brief loss of consciousness | 50 |
| Stable cardiovascular/ medical conditions required; patients with history of autoimmune disorders or seizures excluded | Unstable cardiovascular/medical condition (recent or active heart attack/cardiac arrest, myocardial infarct, congestive heart failure, heart valve replacement/repair, cardiac bypass, angioplasty/endarterectomy/stent, angina, atrial fibrillation, diabetes) | 50 |
| Stable psychiatrically | Unstable psychiatric condition (severe depression/dysphoria; recent/active schizophrenia; recent/active bipolar disorder; severe hallucinations; severe delusions; recent or active alcohol or substance abuse) | 14 |
| Patients with bleeding disorders or on anticoagulants excluded | Use of one of the following medications with anti-coagulant properties (ie, heparin; enoxaparin; dalteparin; danaparoid; ardeparin; tinzaparin; heparin flush; warfarin; anisindione; dicumarol; lepirudin; argatroban; bivalirudin; dabigatran; fondaparinux; rivaroxaban; apixaban) | 8 |
| Patients can be on standard of care with cholinesterase inhibitors and memantine | Participants treated with cholinesterase inhibitors and memantine were not excluded | - |
| Normal serum vitamin B12 level, thyroid stimulating hormone, metabolic panel and liver function tests, complete blood count, comprehensive clotting studies and platelet count. Normal erythrocyte sedimentaton rate and C-reactive protein | Recent/active vitamin B12 deficiency or thyroid disease | 57 |
| Baseline MRI showing  • Acute or subacute hemorrhage  • Macrohemorrhage  • Cortical infarction >1.5 cm  • One lacunar infarction >1.5 cm  • More than four microhemorrhages  • More than one area of superficial siderosis  • Extensive white matter disease indicative of ischemic injury | Evidence of macrohemorrhage(s), microhemorrhage(s), large vessel infarct(s), lacunar infarct(s), extensive white-matter hyperintensity (Cardiovascular Health Study score 7-8) | 0 |
| Patient and care partner must understand the nature and requirements of therapy and the expected outcome of therapy | Co-participant available | 5 |

Operationalization of inclusion and exclusion criteria of the Appropriate Use Recommendations (left column) onto the NACC dataset by the indicated variable (middle column). NA indicates the absence of the respective information in the dataset. For all criteria that could be mapped onto the NACC dataset, the number of participants not fulfilling the respective criteria is indicated (rightmost column). Abbreviations: AUR = Appropriate Use Recommendations; NINDS/AIREN = National Institute of Neurological Disorders and Stroke / Association Internationale pour la Recherche et l'Enseignement en Neurosciences. Note that some criteria of the trial were shortened/ summarized to improve readability.

**Table 3:** Operationalization of the mapping of selection criteria of the lecanemab trial on the NACC dataset

| Trial criteria | Operationalization the NACC database | No. of participants not fulfilling criterion |
| --- | --- | --- |
| **Inclusion criteria** |  |  |
| Positive biomarker for brain amyloid pathology | NA | - |
| 50-90 years of age | Subject’s age at initial visit 50-90 years (inclusive) | 32 |
| Mini Mental State Examination (MMSE) score > 22 at Baseline and < 30 and Baseline | Total MMSE score 22-30 (inclusive) | 390 |
| Body mass index (BMI) greater than (>)17 and less than (<) 35 | BMI 18.0-34.9 kg/m^2^ | 55 |
| If receiving an acetylcholinesterase inhibitor (donepezil, rivastigmine, galantamine) or memantine or both must be on a stable dose for at least 12 weeks prior to Baseline | NA | - |
| Unless otherwise stated, participants must have been on stable doses of all other (that is, non-AD-related) permitted concomitant medications for at least 4 weeks prior to Baseline | NA | - |
| Have an identified study partner | Co-participant available | 3 |
| Provide written informed consent | NA | - |
|  |  |  |
| **Exclusion criteria** |  |  |
| Any neurological condition that may be contributing to cognitive impairment above and beyond that caused by the participant’s AD | Neurological condition primary or contributing cause of cognitive impairment (ie, cerebrovascular disease; vascular brain injury; stroke; probable vascular dementia (NINDS/AIREN criteria); Lewy body disease; multiple system atrophy; primary supranuclear palsy; corticobasal degeneration; frontotemporal lobar degeneration (with motor neuron disease, behavioral variant, or not otherwise specified); primary progressive aphasia; essential tremor; Down syndrome; Huntington’s disease; Prion disease (Creutzfeldt-Jakob disease, other); traumatic brain injury; normal-pressure hydrocephalus; epilepsy; central nervous system neoplasm; human immunodeficiency virus; other neurological, genetic, or infectious condition; delirium) | 34 |
| More than 4 microhemorrhages (defined as 10 millimeter [mm] or less at the greatest diameter); a single macrohemorrhage >10 mm at greatest diameter; an area of superficial siderosis; evidence of vasogenic edema; multiple lacunar infarcts or stroke involving a major vascular territory; severe small vessel; or other major intracranial pathology | Evidence of microhemorrhage(s), macrohemorrhage(s), lacunar infarct(s), large vessel infarct(s), extensive white-matter hyperintensity (Cardiovascular Health Study score 7-8) | 0 |
| Evidence of other clinically significant lesions on brain MRI at Screening that could indicate a dementia diagnosis other than AD | NA | - |
| Recent history of transient ischemic attacks (TIA), stroke, or seizures; any psychiatric diagnosis or symptoms (example, hallucinations, major depression, or delusions) that could interfere with study procedures in the participant; Geriatric Depression Scale (GDS) score > 8 | Recent/active transient ischemic attack, stroke or seizures; psychiatric diagnosis or symptoms (moderate/severe hallucinations; severe depression/dysphoria; moderate/severe delusions; recent/active schizophrenia; recent/active bipolar disorder, Geriatric Depression Score >8) | 27 |
| Any immunological disease which is not adequately controlled, or which requires treatment with immunoglobulins, systemic monoclonal antibodies (or derivatives of monoclonal antibodies), systemic immunosuppressants, or plasmapheresis during the study | NA | - |
| Participants with a bleeding disorder that is not under adequate control (including a platelet count <50,000 or international normalized ratio [INR] >1.5 for participants who are not on anticoagulant treatment, example, warfarin) | NA | - |
| Participants who are on anticoagulant therapy should have their anticoagulant status optimized and be on a stable dose for 4 weeks before Screening | Data on recent change of medication is limited | - |
| Any other medical conditions (example, cardiac, respiratory, gastrointestinal, renal disease) which are not stably and adequately controlled, or which could affect the participant’s safety or interfere with the study assessments | Unstable cardiac condition (recent/active heart attack or cardiac arrest, myocardial infarct, congestive heart failure, heart valve replacement/repair, cardiac bypass procedure, angioplasty/endarterectomy/stent, angina; atrial fibrillation) | 27 |

Operationalization of inclusion and exclusion criteria of the trial (left column) onto the NACC dataset by the indicated variable (middle column). NA indicates the absence of the respective information in the dataset. For all criteria that could be mapped onto the NACC dataset, the number of participants not fulfilling the respective criteria is indicated (rightmost column). Abbreviations: NINDS/AIREN = National Institute of Neurological Disorders and Stroke / Association Internationale pour la Recherche et l'Enseignement en Neurosciences. Note that some criteria of the trial were shortened / summarized to improve readability.

**Table 4:** Operationalization of the mapping of selection criteria for treatment with lecanemab according to the AUR

| Appropriate Use Recommendations criteria | Operationalization the NACC database | No. of participants not fulfilling criterion |
| --- | --- | --- |
| **Inclusion criteria** |  |  |
| Positive amyloid PET or CSF studies indicative of AD | NA | - |
| Physician judgement used for patients outside the 50-90 year age range | Age at initial visit ≤95 years | 5 |
| MMSE 22-30 or other cognitive screening instrument with a score compatible with early AD | Total MMSE score 22-30 (inclusive) | 406 |
| Physician judgement used for patients at the extremes of Body mass index (BMI) | BMI 18.0-40.0 kg/m^2^ | 46 |
| Patients may be on cognitive enhancing agents (donepezil, rivastigmine, galanta­mine, or memantine) for AD; patients may not be on aducanumab | Participants with or without cognitive enhancing agents were included; no information available concerning treatment with aducanumab | 0 |
| Patients may be on standard of care for other medical illnesses (see below for specifics regarding anticoagulation) | Participants with or without standard care for other medical illnesses were included (see below exclusion criteria regarding anticoagulation) | 0 |
| Have a care partner or family member(s) who can ensure that the patient has the support needed to be treated with lecanemab | Co-participant available | 3 |
| Patients, care partners, and appropriate family members should understand the requirements for lecanemab therapy and the potential benefit and potential harm of treatment | NA | - |
|  |  |  |
| **Exclusion criteria** |  |  |
| Any medical, neurologic, or psychiatric condition that may be contributing to the cognitive impairment or any non-AD MCI or dementia | Medical, neurologic, or psychiatric condition primary or contributing cause of cognitive impairment (ie, cerebrovascular disease; vascular brain injury; stroke; probable vascular dementia (NINDS/AIREN criteria); Lewy body disease; multiple system atrophy; primary supranuclear palsy; corticobasal degeneration; frontotemporal lobar degeneration (with motor neuron disease, behavioral variant, or not otherwise specified); primary progressive aphasia; essential tremor; Down syndrome; Huntington’s disease; Prion disease (Creutzfeldt-Jakob disease, other); traumatic brain injury; normal-pressure hydrocephalus; epilepsy; central nervous system neoplasm; human immunodeficiency virus; other neurological, genetic, or infectious condition; delirium; depression; bipolar disorder; schizophrenia or other psychosis; anxiety; posttraumatic stress disorder; other psychiatric disease; alcohol abuse; other substance abuse; systemic disease/medical illness; medications; undetermined etiology) | 58 |
| More than 4 microhemorrhages (defined as 10 mm or less at the greatest diameter); a single macrohemorrhage >10 mm at greatest diameter; an area of superficial siderosis; evidence of vasogenic edema; more than 2 lacunar infarcts or stroke involving a major vascular territory; severe subcortical hyperintensities consistent with a Fazekas score of 3; evidence of amyloid beta-related angiitis; cerebral amyloid angiopathy-related inflammation; or other major intracranial pathology that may cause cognitive impairment | Evidence of microhemorrhage(s), macrohemorrhage(s), lacunar infarct(s), large vessel infarct(s), extensive white-matter hyperintensity (Cardiovascular Health Study score 7-8) | 0 |
| MRI evidence of a non-AD dementia | NA | - |
| Recent history (within 12 months) of stroke or transient ischemic attacks or any history of seizures | Recent/active transient ischemic attack, stroke or seizures | 6 |
| Mental illness (e.g, psychosis) that interferes with comprehension of the requirements, potential benefit, and potential harms of treatment and are considered by the physician to render the patient unable to comply with management requirements  Major depression that will interfere with comprehension of the requirements, potential benefit, and potential harms of treatment; patients for whom disclosure of a positive biomarker may trigger suicidal ideation. Patients with less severe depression or whose depression resolves may be treatment candidates | Mental illness (severe hallucinations, severe depression/dysphoria; severe delusions; recent/active schizophrenia; recent/active bipolar disorder) | 6 |
| Any history of immunologic disease (e.g., lupus erythematosus, rheumatoid arthritis, Crohn’s disease) or systemic treatment with immunosuppressants, immunoglobulins, or monoclonal antibodies or their derivatives | NA | - |
| Patients with a bleeding disorder that is not under adequate control (including a platelet count <50,000 or international normalized ratio [INR] >1.5 for participants who are not on anticoagulant) | NA | - |
| Patients on anticoagulants (coumadin, dabigatran, edoxaban, rivaroxaban, apixaban, betrixaban, or heparin) should not receive lecanemab; tPA should not be administered to individuals on lecanemab | Treatment with anticoagulants (warfarin, dabigatran, rivaroxaban, apixaban, heparin) | 16 |
| Unstable medical conditions that may affect or be affected by lecanemab therapy | Unstable medical condition (recent/active heart attack or cardiac arrest, myocardial infarct, congestive heart failure, heart valve replacement/repair, cardiac bypass procedure, angioplasty/ endarterectomy/stent, angina; atrial fibrillation, diabetes) | 30 |

Operationalization of inclusion and exclusion criteria of the Appropriate Use Recommendations (left column) onto the NACC dataset by the indicated variable (middle column). NA indicates the absence of the respective information in the dataset. For all criteria that could be mapped onto the NACC dataset, the number of participants not fulfilling the respective criteria is indicated (rightmost column). Abbreviations: AUR = Appropriate Use Recommendations; NINDS/AIREN = National Institute of Neurological Disorders and Stroke / Association Internationale pour la Recherche et l'Enseignement en Neurosciences. Note that some criteria of the trial were shortened / summarized to improve readability.

**Table 5:** Operationalization of the mapping of selection criteria of the donanemab trial on the NACC dataset

| Trial criteria | Operationalization the NACC database | No. of participants not fulfilling criterion |
| --- | --- | --- |
| **Inclusion criteria** |  |  |
| 60 to 85 years of age inclusive, at the time of signing the informed consent | Age at initial visit 60-85 years (inclusive) | 181 |
| Gradual and progressive change in memory function reported by the participant or informant for ≥6 months | Time since onset of cognitive decline ≥1 year | 16 |
| MMSE score of 20 to 28 (inclusive) | Total MMSE score 20-28 (inclusive) | 235 |
| Meet flortaucipir/florbetapir F18 scan criteria | NA | - |
| Have a study partner and will accompany the participant to study visits or be available by telephone at designated times | Co-participant available | 7 |
| Have adequate literacy, vision, and hearing for neuropsychological testing | Normal vision (if necessary, with corrective lenses) and normal hearing (if necessary, using hearing aid(s)) | 23 |
| Are reliable and willing to make themselves available for the duration of the study and are willing to follow study procedures | NA | - |
| Stable concomitant symptomatic AD medications and other medication that may impact cognition for at least approximately 30 days prior to randomization | Data on recent change of AD medication is limited | - |
| Contraceptive use by men or women | NA | - |
| Capable of giving signed informed consent | NA | - |
| **Exclusion criteria** |  |  |
| Significant neurological disease affecting the central nervous system other than AD, that may affect cognition or ability to complete the study, including but not limited to, other dementias, serious infection of the brain, Parkinson’s disease, multiple concussions, or epilepsy or recurrent seizures (except febrile childhood seizures) | Neurological condition primary or contributing cause of cognitive impairment (ie, cerebrovascular disease; vascular brain injury; stroke; probable vascular dementia (NINDS/AIREN criteria); Parkinson’s disease; Lewy body disease; multiple system atrophy; primary supranuclear palsy; corticobasal degeneration; frontotemporal lobar degeneration (with motor neuron disease, behavioral variant, or not otherwise specified); primary progressive aphasia; essential tremor; Down syndrome; Huntington’s disease; Prion disease (Creutzfeldt-Jakob disease, other); traumatic brain injury; normal-pressure hydrocephalus; epilepsy; recent/active seizures; central nervous system neoplasm; human immunodeficiency virus; other neurological, genetic, or infectious condition; delirium) | 40 |
| Current serious or unstable illnesses including cardiovascular, hepatic, renal, gastroenterologic, respiratory, endocrinologic, neurologic (other than AD), psychiatric, immunologic, or hematologic disease and other conditions that, in the investigator’s opinion, could interfere with the analyses in this study; or has a life expectancy of <24 months; history of cancer within the last 5 years, with the exception of non-metastatic basal and/or squamous cell carcinoma of the skin, in situ cervical cancer, nonprogressive prostate cancer, or other cancers with low risk of recurrence or spread | Current cardiovascular (recent/active heart attack or cardiac arrest; myocardial infarct present within the past 12 months; recent/active congestive heart failure; recent/active heart valve replacement or repair; recent/active angina; atrial fibrillation; recent/active cardiac bypass procedure; pacemaker (recent/active); recent/active angioplasty/endarterectomy/stent), endocrinologic (recent/active thyroid disease; recent/active diabetes), psychiatric (severe depression/dysphoria; recent/active or remote/inactive schizophrenia; recent/active bipolar disorder; moderate/severe hallucinations; moderate/severe delusions) disease; cancer (primary/non-metastatic or metastatic) | 115 |
| Current primary psychiatric diagnosis other than AD if the psychiatric disorder or symptom is likely to confound interpretation of drug effect, affect cognitive assessment, or affect the participant’s ability to complete the study. Participants with history of schizophrenia or other chronic psychosis are excluded | Psychiatric condition primary or contributing cause of cognitive impairment (depression; bipolar disorder; anxiety; post-traumatic stress disorder; other psychiatric disease; alcohol abuse; other substance abuse) | 12 |
| Participants who are actively suicidal and therefore deemed to be at significant risk for suicide | NA | - |
| Recent history of alcohol or drug use disorder (except tobacco use disorder) | Alcohol abuse – clinically significant impairment occurring over a 12-month period manifested in one of the following areas: work, driving, legal, or social (recent/active); other abused substances – clinically significant impairment occurring over a 12-month period manifested in one of the following areas: work, driving, legal, or social (recent/active) | 4 |
| History of clinically significant multiple or severe drug allergies, significant atopy, or severe posttreatment hypersensitivity reactions | NA | - |
| Have any clinically important abnormality in physical or neurological examination, vital signs, ECG, or clinical laboratory test results that could be detrimental to the participant, could compromise the study, or show evidence of other etiologies for dementia | Abnormal findings in the physical/neurological examination at the initial visit; focal deficits present indicative of central nervous system disorder; blood pressure reading <90 or >180 (systolic)/<50 or >100 (diastolic) mmHg; resting heart rate <40 or >100/min | 44 |
| MRI which shows evidence of significant abnormality that would suggest another potential etiology for progressive dementia or a clinically significant finding that may impact the participant’s ability to safely participate in the study | NA | - |
| Have any contraindications for MRI, including claustrophobia or the presence of contraindicated metal (ferromagnetic) implants/cardiac pacemaker | Participants with pacemaker excluded (see above) | - |
| Have a MRI demonstrating presence of ARIA-E, >4 cerebral microhemorrhages, more than 1 area of superficial siderosis, any macrohemorrhage or severe white matter disease | Evidence of microhemorrhage(s), macrohemorrhage(s), extensive white-matter hyperintensity (Cardiovascular Health Study score 7-8) | 0 |
| Sensitivity to florbetapir/flortaucipir F18 | NA | - |
| Poor venous access | NA | - |
| Contraindication to PET | NA | - |
| Present or planned exposure to ionizing radiation | NA | - |
| Elevated liver proteins | NA | - |
| Have had prior treatment with a passive anti-amyloid immunotherapy; have received active immunization against Aβ in any other study; have known allergies to donanemab; are currently enrolled/have participated in any other interventional clinical trial; have previously received donanemab | NA | - |
| Investigator site personnel directly affiliated with this study, or are Lilly employees or are employees of third-party organizations involved in the study | NA | - |
| Intention to donate blood/blood products | NA | - |

Operationalization of inclusion and exclusion criteria of the trial (left column) onto the NACC dataset by the indicated variable (middle column). NA indicates the absence of the respective information in the dataset. For all criteria that could be mapped onto the NACC dataset, the number of participants not fulfilling the respective criteria is indicated (rightmost column). Abbreviations: NINDS/AIREN = National Institute of Neurological Disorders and Stroke / Association Internationale pour la Recherche et l'Enseignement en Neurosciences. Note that some criteria of the trial were shortened / summarized to improve readability.
